# Growth and adherence of *Staphylococcus aureus* were enhanced through the PGE_2_ produced by the activated COX-2/PGE_2_ pathway of infected oral epithelial cells

**DOI:** 10.1371/journal.pone.0177166

**Published:** 2017-05-04

**Authors:** Yuxia Wang, Biao Ren, Xuedong Zhou, Shiyu Liu, Yujie Zhou, Bolei Li, Yaling Jiang, Mingyun Li, Mingye Feng, Lei Cheng

**Affiliations:** 1 State Key Laboratory of Oral Diseases, Sichuan University, Chengdu, China; 2 Department of Operative Dentistry and Endodontics, West China Hospital of Stomatology, Sichuan University, Chengdu, China; Universidade Federal do Rio de Janeiro, BRAZIL

## Abstract

*Staphylococcus aureus* is a major pathogen of varieties of oral mucous infection. Prostaglandin E2 (PGE_2_) is a pro-inflammatory factor and Cyclooxygenase 2 (COX-2) is a critical enzyme of PGE_2_ biosynthesis. The purpose of this study is to investigate whether *Staphylococcus aureus* can increase PGE_2_ production of oral epithelial cells and how PGE_2_ functions in the growth and adherence of *Staphylococcus aureus*. mRNA levels of COX-2, *fnbpA* and *fnbpB* were estimated by quantitative PCR. PGE_2_ production was measured by Enzyme Linked Immunosorbent Assay (ELISA). The binding biomass of *Staphylococcus aureus* to human fibronectin was investigated by crystal violet staining and confocal laser scanning microscopy and the adherent force was measured by atomic force microscope (AFM). The COX-2 mRNA level and PGE_2_ production were increased by *Staphylococcus aureus*. PGE_2_ promoted the growth and biofilm formation of *Staphylococcus aureus*, enhanced the attachment of *Staphylococcus aureus* to the human fibronectin as well as to the HOK cells. The transcription of *fnbpB* was up-regulated by PGE_2_ in both early and middle exponential phase but not *fnbpA*. These results suggest that the activation of COX-2/PGE_2_ pathway in oral epithelial cell by *Staphylococcus aureus* can in turn facilitate the growth and the ability to adhere of the pathogen. These findings uncover a new function of PGE_2_ and may lead to the potential of COX-2/PGE_2_ targeting in the therapy of inflammation and cancer in both which the COX-2/PGE_2_ pathway were observed activated.

## Introduction

In oral and maxillofacial region, *Staphylococcus aureus* is a common causative agent of the soft tissue infection and jaw osteomyelitis, both of which can hinder patients from normal diet and thus reduce their life quality [1–3]. Especially, the mouth floor cellulitis, a rampant soft tissue infection in oral and maxillofacial with *S*. *aureus* as a main pathogen, can rapidly spread and sometimes develops into life threatening events [4,5]. Additionally, the establishment of the chronic inflammation as a risk factor for carcinogenesis highlights the importance of inflammation prevention and therapy [6,7]. Unfortunately, the routine use of antibiotics in infection therapy often leads to the growing incidence of antibiotic-resistant strains of *S*. *aureus*. Therefore, elucidating the pathogenesis of *S*. *aureus* induced inflammation in oral and maxillofacial becomes essential to better understand and treat the disease.

Prostaglandin E2 (PGE_2_) is an oxygenated metabolite of arachidonic acid. Cyclooxygenase (COX) is a restrict enzyme of PGE_2_ biosynthesis, accounting for the conversion of arachidonic acid to prostaglandin H2 (PGH_2_) which is subsequently catalyzed by PGE synthase into PGE_2_ [8]. So far, three forms of COX have been found, COX-l, COX-2 and COX-3, among which COX-2 expression is inducible and is increased in many cases of inflammation and cancer [9–14]. In head and neck squamous cell carcinoma and in the oral mucosa of active smokers, for example, increased levels of COX-2 expression and PGE_2_ production were detected according to previous reports [15,16]. Although *S*. *aureus* was previously shown to induce PGE_2_ production in some cell lines [17,18], no study on the COX-2 expression in oral epithelial cell suffering from *S*. *aureus* infection has been found so far.

As an essential homeostatic factor, PGE_2_ is generally recognized as a key mediator of immunopathology in chronic infections, regulating many courses of inflammation and multiple functions of some immune cells [19–21]. Accumulated evidence has made the establishment of the paradoxes of PGE_2_ function. On the one hand, it acts as a pro-inflammatory mediator activating neutrophils, macrophages, and mast cells at early stages of inflammation [20–22]. On the other hand, it has been demonstrated to be a potent immunosuppressant suppressing both innate and specific immunity at the molecular and cellular levels [23–28]. It limits the cytolytic effector functions of NK cells [29,30] as well as inhibits the phagocytosis and pathogen-killing by alveolar macrophages [31,32], for instance. The immunosuppression of PGE_2_ makes it a potent risk factor in inflammation. Besides, previous studies suggested that chronic inflammation promotes cancer development through COX-2/PGE_2_ pathway [33,34]. Thus, the functional versatility of PGE_2_ is increasingly noteworthy.

Although increasingly clear vision of the paradoxical role of PGE_2_ in various cells in immune responses, little attention has been given to the effect of PGE_2_ on bacterial pathogen—constitute of the inflammation environment. In *C*. *albicans*, PGE_2_ was demonstrated to induce germ tube formation and involve in biofilm formation [35,36]. Jan Krause et al reported that PGE_2_ from *C*. *albicans* stimulates the growth of *S*. *aureus* in mixed biofilms [37]. These results implied a facilitating effect of PGE_2_ on some pathogens. The pathogenesis of bacteria usually bases on its colonization to host tissues. The ability of *S*. *aureus* to adhere is crucial for its early colonization to host tissue and implanted biomaterials. Fibronectin-binding proteins (FnBPs) mediating the binding of *S*. *aureus* to mammalian extracellular matrix of fibronectin are important for the adherence of *S*. *aureus* in the course of infection [38,39]. *fnbpA* and *fnbpB* are genes coding for FnBPs and contribute to the ability of *S*. *aureus* adhering to fibronectin-coated surfaces [40,41]. Previous studies showed that the colonization of *S*. *aureus* is higher in some infection and cancer tissues which have both been reported to display an inducible COX-2 expression and an increased PGE_2_ production [9,11,42]. However, whether the higher rate of *S*. *aureus* colonization is resulted from the increased PGE_2_ level has not been known. Therefore, studying the effect of PGE_2_ on the adherence of *S*. *aureus* to cells is beneficial to elucidate the causal relationship between *S*. *aureus* colonization and inflammation or caner.

Thus, we here in this study propose that following a challenge with *S*. *aureus*, oral mucosal epithelial cell can increase COX-2 expression and PGE_2_ production and *S*. *aureus* can take advantage of the PGE_2_ to grow and to adhere. To confirm our hypothesis, we investigated the COX-2 mRNA level by qPCR and the PGE_2_ level by ELISA in HOK cell line with or without *S*. *aureus* infection. Also, we investigated the effect of PGE_2_ on *S*. *aureus* growth and adherence. The results indicated that *S*. *arueus* can activate the COX-2/PGE_2_ pathway in HOK and that PGE_2_ can promote the growth and biofilm formation of *S*. *aureus*, facilitate the ability of *S*. *aureus* to fibronectin and to HOK cells, and up-regulate the transcriptional level of *fnbpB* in *S*. *aureus*. Our results uncovered a new function of PGE_2_ in the interaction between *S*. *aureus* and oral epithelial cells in the inflammation, directing a new preventive and therapeutic guide for *S*. *aureus* infection.

## Materials and methods

### Cell line, bacterial strain and culture

The cell line of human oral keratinocyte (HOK) was cultured in high glucose Dulbecco’s modified Eagle Medium (DMEM, Hyclone, Logan, UT, USA) containing L-glutamine (2mM) with 10% Fetal bovine serum (FBS, Gibco, Thermo Fisher Scientific, Inc., Waltham, MA, USA), 1% penicillin–streptomycin antibiotic mixture (PS, Hyclone, Logan, UT, USA). The cells were cultured in an incubator with 5% CO_2_ and 95% air at 37°C. Cells were passaged at regular intervals depending on their growth characteristics using 0.25% trypsin (Hyclone, Logan, UT, USA). *Staphylococcus aureus* strain ATCC 25923 was routinely cultured in Tryptone soya broth (TSB, Oxoid, Basingstoke, UK) and 1.5% agar was added when needed.

For the infection assay, exponential phase *S*. *aureus* was centrifuged at 4000rpm for 15min and washed twice with sterile PBS. The pellet was suspended in fresh DMEM without FBS and PS and the suspension was diluted to the required cell density corresponding to ~1×10^8^ CFUs/mL. HOK was incubated in 6-wells plates for 48h with either 0.025% dimethyl sulfoxide (DMSO) or 20μM NS-398 (Sigma-Aldrich; Saint Louis, Missouri), a specific COX-2 inhibitor, dissolved in DMSO at an optimal dose that was previously determined to provide inhibition of COX-2 [18,43,44]. Then, the NS-398- or DMSO-treated cells were infected with *S*. *aureus* at MOI of 100:1. *S*. *aureus* suspensions and HOK cells without infection were as negative control and wells without HOK incubation but added with DMEM or *S*. *aureus* suspension were as blank control. All the wells were incubated at 37°C, 5% CO_2_ for 45min. the supernatants were collected and filtrated with 0.22μm microfiltration membrane and stored at -80°C for ELISA or supernatant assay. After being washed with PBS, cells were lysed with TRIzol Reagent (Invitrogen, California, USA) and stored at -80°C for RNA extraction.

### RNA extraction and quantitative real-time PCR

To quantify mRNA of COX-2, *fnbpA* and *fnbpB*, total RNA was isolated from ~1×10^6^ HOK cells or from ~5×10^8^ bacterial cells following the instructions provided with TRIzol reagent (Invitrogen, California, USA). Total RNA yield and purity were determined by absorbance at 260 nm and 280 nm using a NanoDrop-2000 spectrophotometer (Thermo Fisher Scientific Inc., Waltham, MA, USA). cDNA was then synthesized using the PrimeScript RT reagent Kit with gDNA Eraser (Takara Clontech, Japan) according to the manufacturer’s instructions. Real-time PCR was performed on a C1000 Touch™ Thermal Cycler instrument (Bio-Rad, Philadelphia, PA, USA) with the SYBR reagent (Takara, Dalian, China) following the manufacturer’s instructions. The amplification was performed according to the reported protocol with some modifications [45]. A 25-μl mixture of 12.5 μL SYBR qPCR Mix (Takara, Dalian, China), 2 μL PCR primers mix (10 μM), 2 μL diluted template cDNA, and 8.5 μL deionized distilled water was prepared for each gene and subjected to 40 cycles of three steps consisting of denaturation at 95°C for 3 min, denaturation at 95°C for 5 s, annealing at 60°C for 30 s, followed by a melt curve started at 65°C to 95°C with an increment of 0.5°C for 5S. Relative fold changes of COX-2 were normalized against glyceraldehyde-3-phosphate dehydrogenase (GAPDH) expression and *fnbpA* and *fnbpB* were normalized against *S*. *aureus* 16S expression. PCR primers used in this study are listed in Table 1. The amplification efficiency and template specificity for each primer pair were verified and all the assays were conducted with each sample in triplicate.

**Table 1:** Primers used in this study.

Genes	PCR primers	Description and product size
*cox-2*	For 5'-TCCTGAAACCCACTCCCAACA-3'	Cyclooxygenase 2
	Rev 5'-TGGGCAGTCATCAGGCACAG-3'	242 bp[46]
GAPDH	For 5'-GTCTTCACTACCATGGAGAAGG-3'	glyceraldehyde-3-phosphate dehydrogenase
	Rev 5'-TCATGGATGACCTTGGCCAG-3'	197bp [46]
*fnbpA*	For 5'-ACCGTCAAACGCAACACAAG-3'	Fibronectin-binding protein A
	Rev 5'-TTCTGATGCCGTTCTTGGCT-3'	259bp
*fnbpB*	For 5'-GCTGCAGCATCGGAACAAAA-3'	Fibronectin binding protein B
	Rev 5'-TGCTTGCACAGTTTTCGGTG-3'	201bp
16S	For 5'-TTGGTCCTGAGGGTGGTTTG-3'	Normalizing internal standard
	Rev 5'-CGCATACAATGGCGCAGTTT-3'	113bp

### PGE_2_ ELISA

The supernatant collection for PGE_2_ measurement was described above. Simply, HOK cells were cocultured with *S*. *aureus* at a MOI of 100:1 in DMEM without FBS for 45 min. the supernatants were harvested, filtrated and the PGE_2_ content was measured by ELISA (Cayman Chemical, Ann Arbor, MI) according to the manufacturer’s instructions with each sample in triplicate [47].

### *Staphylococcus aureus* growth and biofilm formation

Exogenous PGE_2_ (Sigma-Aldrich) was dissolved in absolute ethanol to prepare the stock solution of 10mg/mL and the aliquots were stored at -20°C protected from light. For experiment, the aliquots of stock solution were diluted in sterile PBS to obtain the desired concentrations. The overnight culture of *S*. *aureus* was diluted 1:10 into fresh TSB. Then, PGE_2_ dilutions were added into the diluted *S*. *aureus* cultures at the final concentrations of 0 pg/mL, 20 pg/mL, 50 pg/mL, 100 pg/mL and 500 pg/mL. The mixtures of *S*. *aureus* and PGE_2_ were then transferred into the flat-bottom 96-wells cell culture cluster (Corning, NY, USA). Wells containing equal volume of fresh TSB were used as negative controls. The plates were incubated at 37°C aerobically for 18 h. For the planktonic growth measurement, the plate was incubated in the BioTek microplate reader (Gene Company, American) with shaking per 5 seconds and reading per hour. For the biofilm assay, *S*. *aureus* was cultured in TSB medium (containing 5% FBS) added with PGE_2_ (500 pg/mL) or with PBS and then the culture was transferred into the flat-bottom 96-well cell culture cluster as described above. The plates for biofilm formation were incubated at 37°C statically and after 18h incubation, Liquid medium was removed and the wells were gently rinsed two times with sterile distilled water to remove the planktonic or loosely bound cells. Biofilms were stained with crystal violet (CV) solution as described by Peeters *et al* with a few modifications [48]. Briefly, biofilms cultured in 96-well plates were fixed with glutaraldehyde. Then, 50 μL of 0.1% crystal violet solution was added to each well and incubated for 20 min at room temperature. Excess CV was removed by washing under running tap water and bounded CV was released by 200 μL of 99% ethanol. Absorbance was measured at 570 nm with a Thermo Scientific Multiskan GO reader (Thermo Fisher Scientific Inc., Waltham, MA, USA)

### Fibronectin assay

Binding of *S*. *aureus* to solid-phase fibronectin was measured as described previously [49,50] with some modification. Briefly, flat-bottomed polystyrene 96-well plates (Corning, NY, USA) were coated with 100 μL fibronectin (10 μg/mL in PBS) isolated from human plasma (Sigma-Aldrich) for 1 h at 37°C. Fibronectin solution was removed and replaced with a blocking solution of Bovine Serum Albumin (BSA, 2%, w/v) in PBS overnight at 4°C. Wells were washed three times with sterile PBS prior to the addition of bacterial suspension. The overnight *S*. *aureus* culture was diluted with fresh TSB that was added with PGE_2_ (500 pg/mL) or added with PBS at the same volumes. Then, the condition dilutions were cultured to the exponential phase. The cultures were centrifuged at 4°C, 4000 rpm for 15 min. The pellets were washed three times and resuspended with PBS. After adjustment to achieve a similar initial cell density, the cultures were diluted 1:10 with DMEM without FBS and antibiotics. 200μL (approx. 2×10^6^ bacteria) of the diluted cells were transferred in quadruplicate into the wells coated with fibronectin and, as a negative control, into the uncoated wells blocked with BSA. Plates were incubated at 37°C for 1 h. All wells were rinsed three times with PBS to remove unbound bacteria. Adherent bacteria were fixed with glutaraldehyde [200 μL; 2% (v/v) in PBS] for 1 h at room temperature. After rinsing, bacteria in 96-well microtitre plates were stained with 50 μL crystal violet (final concentration 0.01%, w/v) for 15 min. Wells were rinsed three times with sterile water and allowed to air dry. 200 μL 99% ethanol was added to each experimental well and the plates were shaken for several minutes to induce dye release. The quantity of biomass was represented by OD_570_ measured with a Thermo Scientific Multiskan GO Reader.

### Confocal laser scanning microscopy for *S*. *aureus* adhesion to fibronectin

A flat-bottom 24-well plate with cover glasses in the bottoms was coated and blocked by the protocol described above for the fibronectin assay. The prepared bacterial suspensions pre-cultured with PGE_2_ at 0pg/mL or 500pg/mL were added into the prepared wells. The plate was cultivated at 37°C aerobically for 1 h and 3 h without agitation. The glass slides were dyed with a SYTO-9 staining (Molecular Probes, Eugene, OR, USA). After incubation at room temperature in the dark for 15 min, the samples were fixed with mounting oil that protected against fluorescence quenching, immobilized by nail polish on slides and stored at 4°C away from the light before being scanned by a Leica TCS SP2 confocal laser scanning microscopy (CLSM, Leica, German). All the samples were observed by an oil lens and images were at the same magnification of 630×. The excitation/emission for scanning were 480nm/500nm respectively following the instruction and the interval was 1 μm. Images were recorded from signal appeared to signal disappeared and analyzed by a LAS AF Lite software without zoom in.

### Measurement of adhesion forces

The cover glasses were coated with human fibronectin as protocol described in the CLSM assay and slides without fibronectin coated were used as control. The preparation of bacterial AFM tips and the measurement of adhesion forces were followed the protocol from study by Chuanyong Wang et al [51]. Briefly, the overnight *S*. *aureus* culture was diluted and incubated in fresh TSB broth with (500 pg/mL) or without PGE_2_ to exponential phase. Then the cultures were centrifuges at 4°C 4000rpm for 15 min and washed twice with PBS and resuspended in adhesion buffer (2 mM potassium phosphate, 50 mM potassium chloride, 1 mM calcium chloride; pH 6.8) [52]. The bacterial suspensions were then sonicated 3 times (10 s working with 5 s waiting each) to scatter bacteria clumps. The CSC38/tipless AFM probe (Ultrasharp, m-Masch, Tallinn, Estonia) were sterilized under UV for 5 min and the half length of the cantilever was dipped into a drop of 0.01% (w/v) poly-L-lysine (Sigma, Poole, UK) for 1 min. After being dried for 2 min in air, the probes were immersed in bacterial suspension for 1 min and were applied for adhesion force measurement immediately. For each probe, two coated and two un-coated slides were measured with 6–8 positions being randomly selected for every slide and each position being repeated at least ten times. Each sample was tested with at least three tips. Every time after used, the tip was detected under a Scanning electron micrograph (SEM) to confirm the intactness of the bacteria layer on the modified cantilever. The force data was disposed once the integrity of bacterial layer was damaged.

### Adherent and invasion assay

The adherent and invasion assay was carried out as previously described with some modification [18,53]. Briefly, the suspension of exponential *S*. *aureus* in DMEM was prepared as the protocol for the infection assay. The completely confluent cell layers in 24-well plates were rinsed twice with sterile PBS and added with *S*. *aureus* suspension for each well at the MOI of 100:1. Equal volumes of *S*. *aureus* suspensions were simultaneously added into wells without cells to monitor the growth of *S*. *aureus* in DMEM in the experimental periods. Following 45 min cultured, the *S*. *aureus* was ten-time step diluted and the dilutions were plated on the TSA agar plates and the HOK cells were washed three times with sterile PBS. For adherent assay, 1 mL of 0.1% Triton X-100 (Amresco, Solon, OH, USA) was added into each well and incubated for 5 min at 37°C to lyse the cells. The lyses were ten-time step diluted and the dilutions were plated on the TSA ager plates. Meanwhile, for the invasion assay, other cells were incubated in DMEM containing gentamicin (100 μg/mL) for 1 h to kill the remaining extracellular bacterial cells and then the cells were lysed and plated after rinsed as the protocol described above for the adherent assay. The plates were incubated at 37°C for 24 h and the single clones were calculated.

### The supernatant assay

HOK cells were pretreated with 20 μM NS-398 or with 0.025% DMSO for 48 h and then infected with *S*. *aureus* at MOI of 100:1. Cells uninfected were as negative control and DMEM and *S*. *aureus* suspension were as blank control. All the wells were incubated at 37°C, 5% CO_2_ for 45min and the supernatants were collected by centrifuged and filtrated. The overnight culture of *S*. *aureus* was diluted with fresh TSB. Then, the diluted cultures were added with the conditional cell supernatants at the final concentration of 20% or added with PGE_2_ at the concentration of 136.5 pg/mL which equals to the quantity of PGE_2_ in the supernatants added. After incubated to the exponential phase, the cultures were ten-time step diluted, plated on the TSA agars and incubated at 37°C for 24 h. Or, the cultures were centrifuged at 4000 rpm for 15 min at 4°C, rinsed with sterile PBS and suspended in fresh DMEM. After being adjusted to the same density, the *S*. *aureus* suspensions were added into the 96-well plates coated with fibronectin (1 μg per well) for the fibronectin assay or added into 24-well plates with confluent HOK layers for the adherent assay as described above.

### Statistical analysis

All experiments were performed in triplicate and results are representative of at least three independent experiments. Comparisons between groups were analyzed by analysis of variance (ANOVA) unless otherwise stated. Data are expressed as means ± SE and results were considered to be statistically significant where *P* < 0.05.

## Results

### Induction of COX-2 mRNA expression and PGE_2_ production in HOK after exposure to *S*. *aureus*

**Fig 1:**
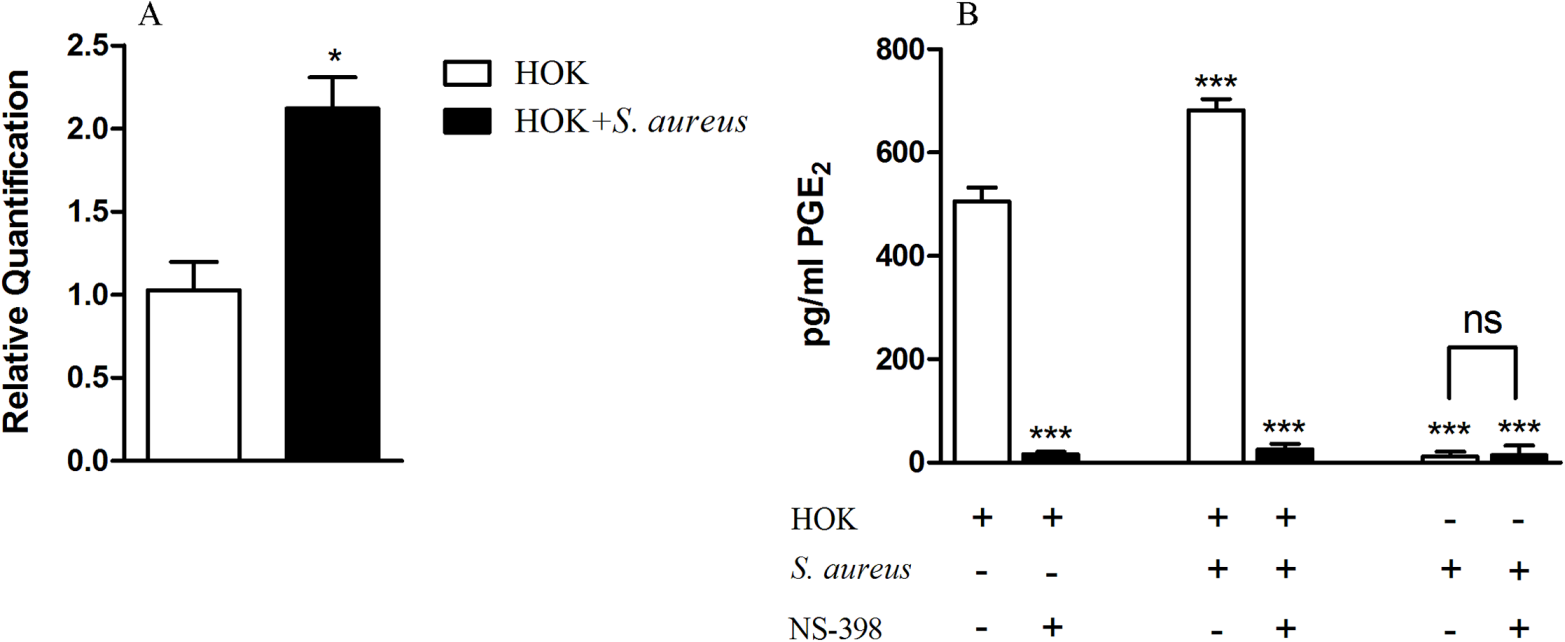
*S*. *aureus* increases the expression of COX-2 mRNA and PGE_2_ production by HOK. HOK cells were pretreated with 20μM NS-398 or with 0.025% DMSO for 48 h. Then, the cells were infected with *S*. *aureus* at the MOI of 100:1 for 45 min. *S*. *aureus* suspension and the HOK without infection were as negative control and DMEM as the blank control. After the infection, the supernatants were collected by centrifuging at 2000 rpm for 25 min and filtrating with 0.22 μM filter membrane and used for PGE_2_ ELISA. The conditional cells were lysed with TRIzol reagent and used for RNA extraction and qPCR. ***A*.** Fold changes of COX-2 mRNA. The level of COX-2 mRNA in *S*. *aureus*-infected HOK doubles that in HOK without infection. ***B*.** The quantity of PGE_2_. The PGE_2_ production by the *S*. *aureus*-infected HOK was 682 pg/mL, significantly higher than that by the un-infected HOK which was 505 pg/mL. Data are expressed as means ± standard errors from three independent experiments and asterisks represent significant differences (*P* < 0.05) compared with HOK.

Previous studies showed that *S*. *aureus* can induce PGE_2_ production in nasal fibroblasts and murine osteoblasts [17,18]. To investigate whether COX-2 expression in normal oral epithelial cell can be induced by *S*. *aureus* infection, we quantified COX-2 mRNA by qPCR and PGE_2_ production by ELISA with HOK cell line as a model of normal oral epithelium. As shown in Fig 1, the level of COX-2 mRNA in *S*. *aureus*-infected HOK doubles that in HOK without infection (Fig 1A). In accordance with gene regulation, the PGE_2_ production by the *S*. *aureus*-infected HOK was significantly higher than that by the un-infected HOK, 682 pg/mL and 505 pg/mL respectively, that is 35% higher for the infected *S*. *aureus* than the un-infected one (Fig 1B).

Considering that COX-2 is not the only restrict enzyme for PGE_2_ production, we additionally determined whether *S*. *aureus*-stimulated increase of PGE_2_ production was COX-2 derived. By adding NS-398, a small-molecule specific inhibitor of COX-2, into the culture of HOK with or without *S*. *aureus* infection, we found that the level of PGE_2_ both decreased remarkably and the *S*. *aureus*-stimulated increase of PGE_2_ production by HOK infection was disappeared, displaying a similarly low level to that in control cell (Fig 1B). Collectively, these results indicate that *S*. *aureus* up-regulates COX-2 transcription which subsequently leads to PGE_2_ production increased in infected HOK.

### Changes in the growth and biofilm formation of *S*. *aureus* after treatment with PGE_2_

**Fig 2:**
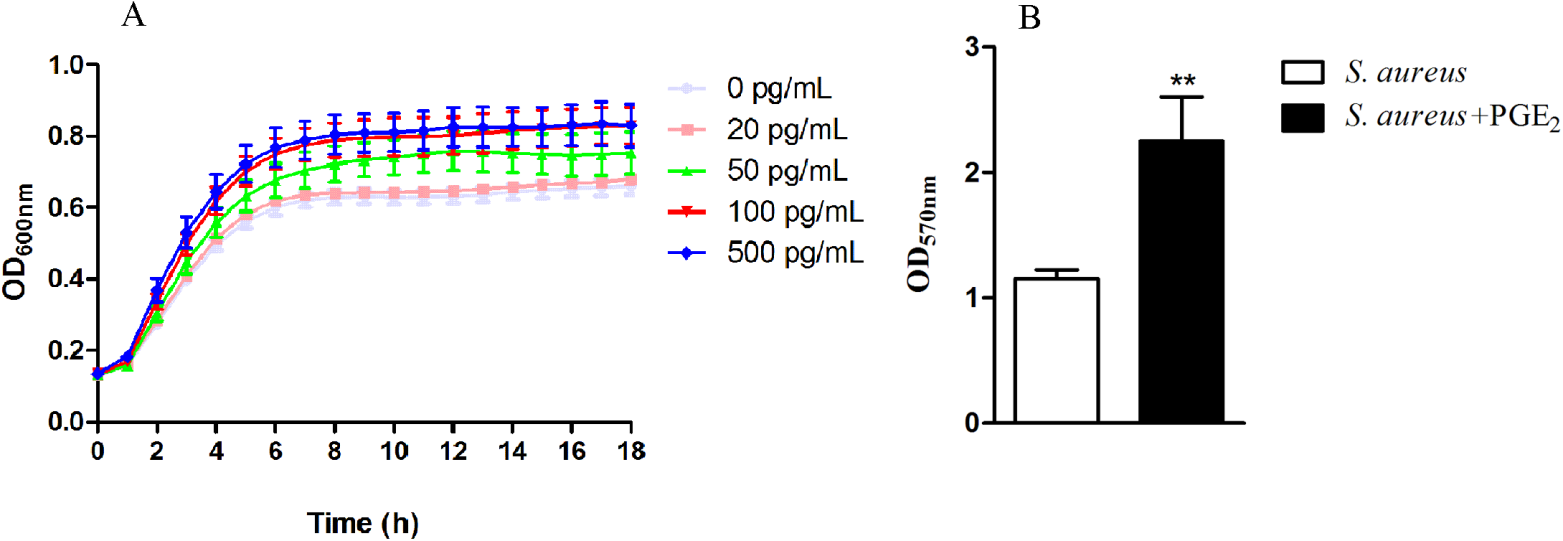
PGE_2_ stimulates the growth and biofilm formation of *S*. *aureus*. *S*. *aureus* was cocultured with PGE_2_ at the final concentrations of 0 pg/mL, 20 pg/mL, 50 pg/mL, 100 pg/mL and 500 pg/mL in 96-well plates for 18 h and the growth curves were recorded by the BioTek microplate reader. Or, *S*. *aureus* cultured in TSB containing 5% FBS was incubated with PGE_2_ (500 pg/mL) or with PBS for 18 h and the biofilms were quantified with 0.1% (w/v) crystal violet staining. ***A*.** The growth curves of planktonic *S*. *aureus* with or without PGE_2_ treatment. PGE_2_ facilitated the growth of *S*. *aureus* in a dose-dependent manner. The facilitated effect of PGE_2_ on *S*. *aureus* growth was observed at the concentration of 50 pg/mL compared with the control and the facilitation was more significant at the concentration of 100 pg/mL, 200 pg/mL and 500 pg/mL. ***B*.** The biofilms quantified by crystal violet staining and measured at the optical density of 570 nm. Biofilm formation of *S*. *aureus* in presence of PGE_2_ was twice as that by *S*. *aureus* in absence of PGE_2_. Results are expressed as means ± standard errors from three replicates per experiment. Asterisks indicate significant (*P*< 0.05) differences compared to HOK without PGE_2_ treatment.

To investigate the impact of PGE_2_ on *S*. *aureus*, we first studied the growth and biofilm formation of *S*. *aureus* in presence of different concentration of purified PGE_2_. As shown in Fig 2A, *S*. *aureus* in presence of 20pg/mL PGE_2_ has a similar growth rate to the control. However, the growth rate of *S*. *aureus* treated with 50pg/mL PGE_2_ was higher than that of the control and the increase in the growth rate was more significantly in *S*. *aureus* treated with PGE_2_ at the concentration of 100 pg/mL and 500 pg/mL. The results indicated that PGE_2_ facilitates *S*. *aureus* growth in a dose-dependent manner.

Biofilm is the main form in which bacterial exist and function. Thus, we investigated the effect of PGE_2_ on the biofilm formation of *S*. *aureus*. Consistent with the facilitation to the growth in planktonic state, similar increase effect by PGE_2_ was observed on the biofilm formation of *S*. *aureus*. Biofilm formation of *S*. *aureus* in presence to PGE_2_ was twice as that by *S*. *aureus* in absence to PGE_2_ (Fig 2B). These results indicated that PGE_2_ exerts a facilitated effect on *S*. *aureus* growth and biofilm formation.

### Changes in the attachment of *S*. *aureus* to human fibronectin after treatment with PGE_2_

**Fig 3:**
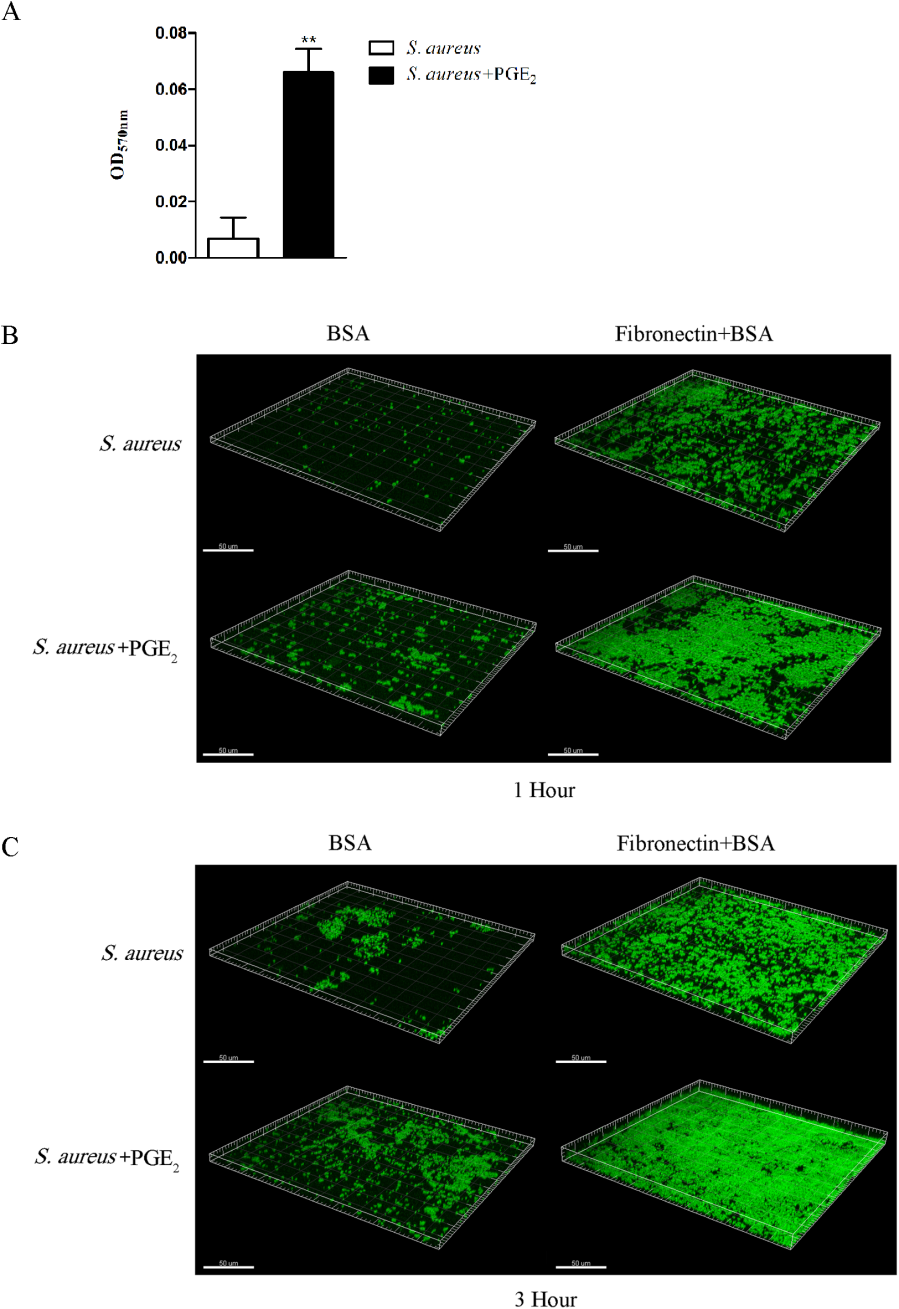
PGE_2_ facilitates the attachment of *S*. *aureus* to human fibronectin. The fibronectin, was coated at 1μg per well in 96-well plates and 5μg per well in 24-well plates set with cover slips at 37°C for 1h, with uncoated wells and slips as the blank control. Then, plates and cover slips were blocked overnight at 4°C. Exponential *S*. *aureus* pretreated with PGE_2_ (500 pg/mL) or PBS was incubated in the conditional 96- or 24-well plates at 37°C. The adherent biomass of *S*. *aureus* was estimated by CV staining at 1 hour after incubation or by CLSM at 1 hour and 3 hour after incubation. ***A*.** The biomass of attached *S*. *aureus* quantified with crystal violent staining and expressed as the optical density at 570 nm. The adherent biomass of *S*. *aureus* cocultured with PGE_2_ was approximately 6.3-fold increased than that by *S*. *aureus* without PGE_2_ treated. Data are expressed as means ± standard errors from three replicates per experiment. Asterisks indicate significant (*P*< 0.05) differences compared to HOK without PGE_2_. ***B*.** The confocal laser scanning microscopy for attached *S*. *aureus* biomass after incubation for 1 hour. ***C*.** The confocal laser scanning microscopy for attached *S*. *aureus* biomass after incubation for 3 hours. At both 1 h and 3 h time points, slides coated with fibronectin displayed higher fluorescent dense intensity than the uncoated ones and, no matter fibronectin treatment or not, the slides were attached with PGE_2_-treated *S*. *aureus* more than the untreated ones. Samples were observed by an oil lens and images were at the same magnification of 630× and analyzed by the LAS AF Lite software without zoom in. The experiments were performed in triple and three images were randomly captured from each sample.

The adherence of *S*. *aureus* to cells is one of its pathogenic factors. Previous studies showed that the binding of cell-wall ligands of *S*. *aureus* to fibronectin plays a major role in the cause of adherence and invasion [38–41]. Therefore, fibronectin assay was performed to investigate whether PGE_2_ has an effect on the attachment of *S*. *aureus* to human fibronectin. 96-well plates and the cover slips in 24-well plates were coated with fibronectin, at 1μg per well for 96-well plates and 5μg per well for 24-well plates, at 37°C for 1h with uncoated wells and slips as the blank control. Then, plates and cover slips were blocked overnight at 4°C. Exponential *S*. *aureus* pretreated with PGE_2_ (500 pg/mL) or PBS was incubated in the conditional 96- or 24-well plates at 37°C. The adherent biomass of *S*. *aureus* was estimated by CV staining at 1 hour after incubation or by CLSM at 1 hour and 3 hour after incubation. As shown in Fig 3A, compared to *S*. *aureus* without PGE_2_ stimulation, the adherent biomass of *S*. *aureus* cocultured with PGE_2_ was significantly more, approximately 6.3-fold increased. Additionally, CLSM assay confirmed the result of CV quantification. As shown in Fig 3B and 3C, after 1 hour or 3 hour cultured, the fluorescent *S*. *aureus* on the slides coated with fibronectin showed higher density than that on the slides without fibronectin, confirming the mediated role of fibronectin in the binding between *S*. *aureus* and cells. Additionally, the fluorescent dense intensity on the slides by *S*. *aureus* cocultured with PGE_2_ was higher than that by *S*. *aureus* in the absence of PGE_2_ both at the 1 hour and at the 3 hour time points, no matter with or without fibronectin treatment. The CV and CLSM results indicated that PGE_2_ facilitates the adherence of *S*. *aureus* to fibronectin.

### Changes in the adhesion force of *S*. *aureus* to human fibronectin after PGE_2_ treatment

**Fig 4:**
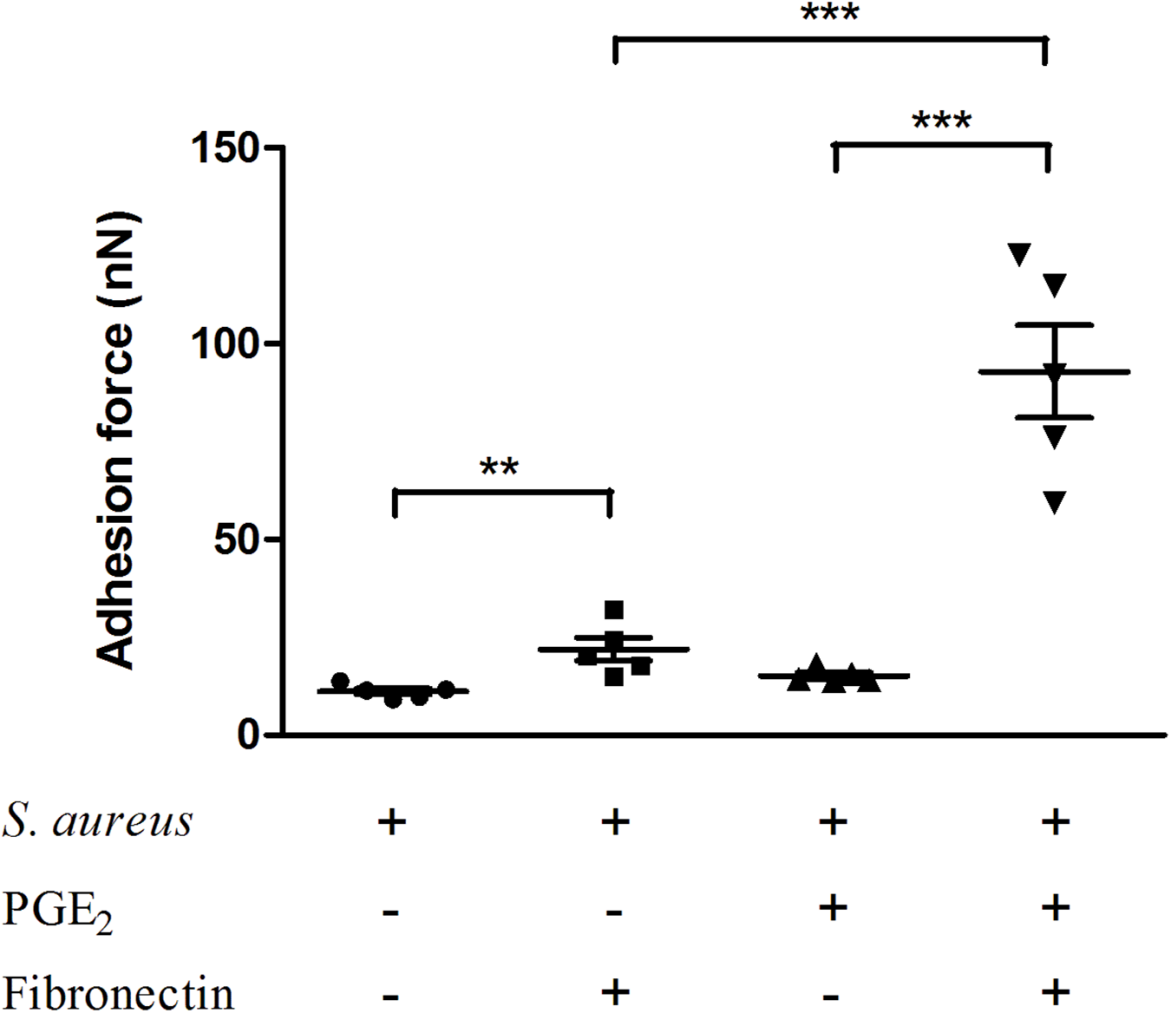
PGE_2_ enhances the adhesion force of *S*. *aureus* to human fibronectin. Exponential *S*. *aureus* cocultured with PGE_2_ (500 pg/mL) or with PBS was harvested and resuspended in adhesion buffer. The CSC38/tipless AFM probes were coated with 0.01% (w/v) poly-L-lysine for 1min, dried in air for 2min, and then immersed in bacterial suspension for 1 min. The adhesion force of *S*. *aureus* to fibronectin or to blank slides was immediately measured by AFM. For *S*. *aureus* without PGE_2_ treatment, the mean adhesion force to the un-coated slides was 11.19 nN and to the fibronectin-coated slides was 21.99 nN. For the PGE_2_-treated *S*. *aureus*, the mean adhesion force to the un-coated slide was 15.24 nN and to the coated slides was 92.87 nN. The adhesion force of *S*. *aureus* to fibronectin was stronger than that to the smooth glass slide surface and PGE_2_ significantly enhanced the force to fibronecin. Each sample was tested with at least three tips and for each probe, two slides were measured. The force data from tip with the integrity of bacterial layer damaged was disposed. Data are expressed as means ± standard errors from three independent experiments and asterisks represent significant differences (*P* < 0.05).

Cover slips were coated or not coated with fibronectin. CSC38/tipless probes were pretreated with 0.01% (w/v) poly-L-lysine and attached with *S*. *aureus* treated or untreated with PGE_2_ (500pg/mL). The adhesion force of *S*. *aureus* to human fibronectin or to blank slides was immediately measured by AFM. As shown in Fig 4, the adhesion forces to the control glasses without fibronectin coated are not different between the PGE_2_ treated and un-treated *S*. *aureus*. Both of the two groups displayed a low force value, that is, 11.19 nN for un-treated *S*. *aureus* and 15.24 nN for PGE_2_-treated *S*. *aureus* respectively. However, when the cover glasses were coated with human fibronectin, the adhesion force of either un-treated *S*. *aureus* or PGE_2_-treated *S*. *aureus* was significantly stronger as compared to their respective control uncoated-glasses, confirming the promotion of fibronectin to *S*. *aureus* adhesion force. Notably, the adhesion force of *S*. *aureus* with PGE_2_ treatment (92.87 nN) is remarkably higher than that of all other groups. For *S*. *aureus* without PGE_2_ treatment, although the adhesion force was markedly enhanced by fibronectin (from 11.19 nN of the control group to 21.99 nN of the fibronectin-coated group), the force value is much less than the 92.87 nN of PGE_2_-treated *S*. *aureus*, indicating the significant enhancement of PGE_2_ to the adhesion force of *S*. *aureus* to human fibronectin.

### Changes in the mRNA levels of *fnbpA* and *fnbpB* after PGE_2_ treatment

**Fig 5:**
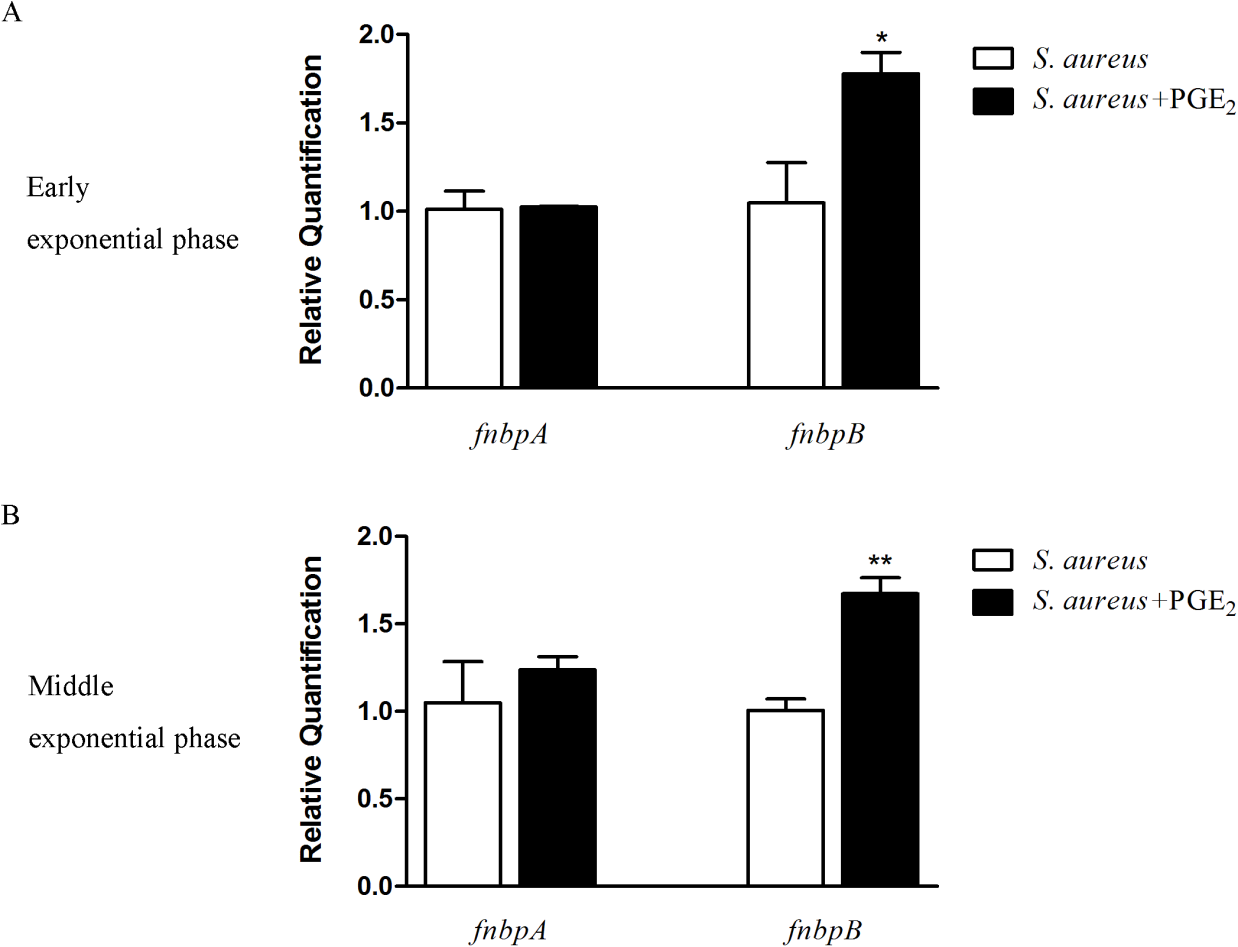
PGE_2_ up-regulates the expression of *fnbpB* but not *fnbpA* mRNA. *S*. *aureus* were treated with PGE_2_ (500 pg/mL) or with PBS and bacterial cells were harvested at both the early and the middle exponential phase. Total RNA of *S*. *aureus* was isolated from ~5×10^8^ bacterial cells using TRIzol reagent following the manufacturer’s instruction. cDNA was synthesized using the PrimeScript RT reagent Kit and qPCR was performed with the SYBR reagent. ***A*.** The transcriptional level of *fnbpA* and *fnbpB* mRNA in the early exponential phase. The level of *fnbpB* mRNA of PGE_2_-treated *S*. *aureus* is 73% higher than that of un-treated *S*. *aureus* and the level of *fnbpA* mRNA had no differences between the two groups. ***B*.** The transcriptional level of *fnbpA* and *fnbpB* mRNA in the middle exponential phase. The level of *fnbpB* mRNA of PGE_2_-treated *S*. *aureus* is 67% higher than that of un-treated *S*. *aureus* and the level of *fnbpA* mRNA had no differences between the two groups. All values were normalized against *S*. *aureus* 16S rRNA expression. Results are representative of three independent experiments and represent the means ± standard errors for three separate cultures. The asterisk represents significant differences (*P* < 0.05) compared with the control *S*. *aureus* that was not pre-cultured with PGE_2_.

To investigate how PGE_2_ facilitates the adherent ability of *S*. *aureus*, we further examined the transcriptions of *fnbpA* and *fnbpB*, two genes coding for the fibronectin-binding proteins that mediate the binding of *S*. *aureus* to the mammalian fibronectin. As shown in Fig 5, in both the early and the middle exponential phase, the transcriptional level of *fnbpA* was not significantly different between the control *S*. *aureus* and PGE_2_-treated *S*. *aureus*. However, the *fnbpB* transcription level of PGE_2_-treated *S*. *aureus* is much higher than that of *S*. *aureus* without PGE_2_ treatment. In detail, the transcriptional level of *fnbpB* of PGE_2_-treated *S*. *aureus* is 73% higher than that of un-treated *S*. *aureus* in the early exponential phase (Fig 5A) and 67% higher in the middle exponential phase (Fig 5B), indicating that PGE_2_ up-regulated *fnbpB* but did no regulation to *fnbpA*.

### Changes in the adherence and invasion of *S*. *aureus* to HOK after PGE_2_ treatment

**Fig 6:**
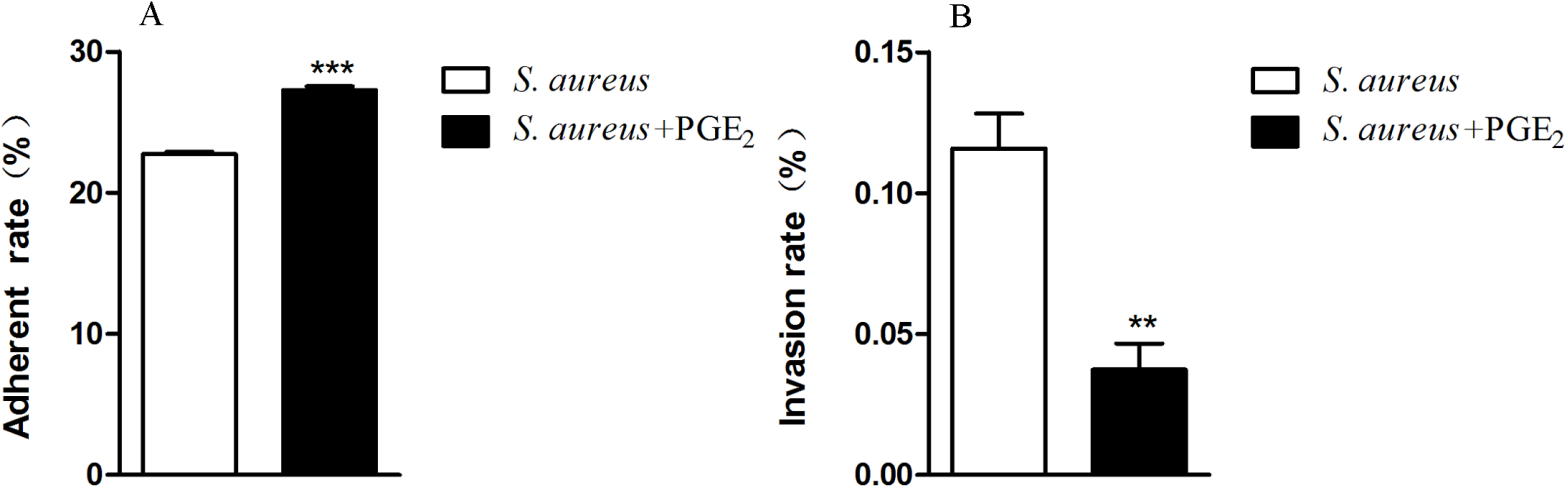
PGE_2_ enhances the adherence but inhibits the invasion of *S*. *aureus* to HOK. Exponential *S*. *aureus* treated with PGE_2_ (500 pg/mL) or PBS was used to infect HOK cells at a MOI of 100:1 for 45 min. Then, the cells were lysed with 1 mL of 0.1% Triton X-100 at 37°C for 5 min, ten-time step diluted and plated on the TSA agar plates. Or, the cells were treated with gentamicin (100 μg/mL) for 1 h and then lysed and plated as above. ***A*.** The adherence rate of *S*. *aureus* to HOK cells. The adherent rate of PGE_2_-stimulated *S*. *aureus* is 27%, significantly higher than the 23% of the control. ***B*.** The invasion rate of *S*. *aureus* to HOK cells. The invasion rate of PGE_2_-treated *S*. *aureus* is lower than the control.The invasive *S*. *aureus* was represented as the number of *S*. *aureus* in lyses of gentamicin-treated cells and the adherent *S*. *aureus* was represented as the difference between *S*. *aureus* in lyses of cells without gentamicin killing and that in lyses of gentamicin-treated cells. The adherence rate and invasion rate were respectively expressed as the ratio of adherent *S*. *aureus* to the total number of incubated *S*. *aureus* and the ratio of invasive *S*. *aureus* to the total number of incubated *S*. *aureus*. Data are expressed as means ± standard errors from three independent experiments and asterisks represent significant differences (*P* < 0.05) compared with *S*. *aureus* without PGE_2_ preculture.

Based on the results of fibronectin assay, we further investigated whether PGE_2_ also has a facilitated effect on the adherence and invasion of *S*. *aureus* to HOK cells. *S*. *aureus* precultured with or without PGE_2_ were adjusted to the same CFUs and cocultured with the conflucent HOK cells at a MOI 100:1 for 45min. Cells with or without gentamicin treatment were then lysed with triton X-100 and the lysate was ten-time step diluted with PBS and plated on the TSA plates. As shown in Fig 6A, the adherent rate of PGE_2_-stimulated *S*. *aureus* is significantly higher than that of the control without PGE_2_ stimulation, 27% and 23% respectively. Inconsistently, the invasion rate of *S*. *aureus* in presence of PGE_2_ is lower than that in absence of PGE_2_, implying multiple layer of PGE_2_ functions at different stages of *S*.*aureus* infection to HOK cells under a fine regulatory mechanism (Fig 6B). Collectively, the results of the adherent assay indicated that PGE_2_ facilitates *S*. *aureus* to adherent to HOK cells.

### The confirmation for the effect of PGE_2_ on *S*. *aureus* with cell supernatants

**Fig 7:**
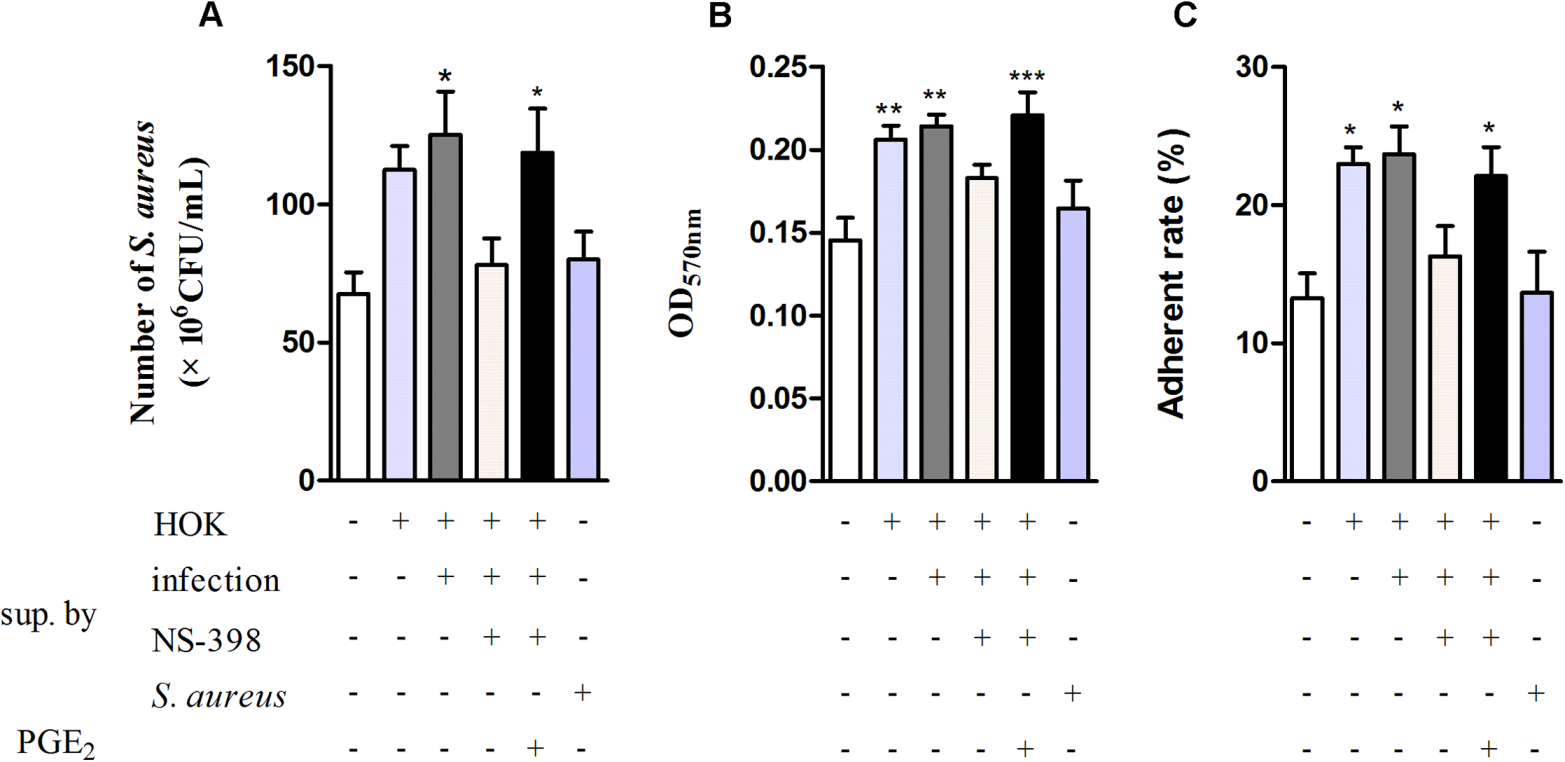
Supernatants containing PGE_2_ produced by HOK facilitate the growth and adherence of *S*. *aureus*. NS-398- or DMSO-treated HOK cells were infected with *S*. *aureus* for 45 min at a MOI of 100:1, with the uninfected cells as negative control and DMEM and *S*. *aureus* suspension as blank controls. Then, the supernatants were collected and used, at the final concentration of 20%, for the stimulation of *S*. *aureus*. Or, exogenous PGE_2_ was added into *S*. *aureus* cultures at the concentration of 136.5 pg/mL which equals to the quantity of PGE_2_ in the infected HOK supernatant added. The growth and adhesion rate to HOK of *S*. *aureus* were evaluates with CFU count and the attachment of *S*. *aureus* to fibronectin was estimated by CV staining. Supernatants are abbreviated to “sup.” in the shown figures. ***A*.** The effect of supernatants on the growth of *S*. *aureus*. Sup-H and Sup-Hi can increase the growth of *S*. *aureus* but Sup-S and Sup-Hi398 can’t. The addition of PGE_2_ recovered the promoted effect of Sup-Hi398, which was inhibited by NS-398, on *S*. *aureu*s growth. ***B*.** The effect of supernatants on the adherence of *S*. *aureus* to fibronectin. The attached biomass of *S*. *aureus* incubated with Sup-H and Sup-Hi was more than that of untreated *S*. *aureus*. Sup-S and Sup-Hi398 had no effect on the attachment of *S*. *aureus* to fibronectin. However, the addition of PGE_2_ into Sup-Hi398 made it regain the facilitated effect on the attachment *S*. *aureus* to fibronecin. ***C*.** The effect of supernatants on the adherence of *S*. *aureus* to HOK cells. The adhesion rates of *S*. *aureus* simulated with Sup-H and Sup-Hi were ~23% and 24% respectively, higher than that of the untreated *S*. *aureus* (~13%). *S*. *aureus* incubated with Sup-S and Sup-Hi398 displayed similar adhesion rates, ~16% and ~14% respectively, to the untreated *S*. *aureus*. The addition of PGE_2_ made the Sup-Hi398 regain the facilitated effect on the adherence of *S*. *aureus* to HOK cells. Sup-H: supernatant from untreated HOK; Sup-Hi: supernatant from infected HOK without NS-398 treated; Sup-Hi398: supernatant from HOK treated with NS-398 and infection. Sup-S: supernatant from *S*. *aureus* suspension. Data are expressed as means ± standard errors from three independent experiments and asterisks represent significant differences (*P* < 0.05) compared with *S*. *aureus* without any supernatant added.

As confirmed above, *S*. *aureus* infection induces PGE_2_ production by HOK and the purified PGE_2_ displayed a facilitated role not only in the planktonic growth but also in the adherence of *S*. *aureus*. To further investigate whether the produced PGE_2_ by HOK has similar facilitated effect on *S*. *aureus*, we subsequently performed the supernatant assay. NS-398- or DMSO-treated HOK cells were infected with *S*. *aureus* for 45 min at a MOI of 100:1 with uninfected cells as negative control and DMEM and *S*. *aureus* suspension as blank control. Then, the supernatants were collected and used, at the final concentration of 20%, for stimulating *S*. *aureus*. Or, exogenous PGE_2_ was added into *S*. *aureus* cultures at the concentration of 136.5 pg/mL which equals to the quantity of PGE_2_ in the infected HOK supernatant. Results from the growth assay by the method of CFUs count revealed that CFUs of *S*. *aureus* in presence of the supernatants from uninfected HOK or treated with the supernatants from infected HOK were significantly more than that of the control. And *S*. *aureus* treated with supernatants from infected HOK displayed the highest growth rate than others. However, when HOK cells were pretreated with NS-398, the supernatants displayed no effect on *S*. *aureus* growth. Notably, the increase in growth rate of *S*. *aureus* was recovered by adding the same concentration of exogenous PGE_2_ into *S*. *aureus* cultures. These results indicated that the COX-2 derived PGE_2_ produced by HOK can promote the growth of *S*. *aureus* (Fig 7A).

Additionally, the effects of the supernatants on the adherence of *S*. *aureus* to fibronectin and HOK cells were also investigated. As shown in Fig 7B and 7C, supernatants from HOK with or without *S*. *aureus* infection both enhanced the adherent rate of *S*. *aureus*, and the infected HOK supernatant displayed more significant promotion than the uninfected HOK supernatant to *S*. *aureus* adherence. Also, the supernatants from NS-398-treated cells had no effect on the adherent of *S*. *aureus* to both fibronectin and HOK cells. However, the facilitated effect was reversed when the NS-398-treated cells supernatants were supplemented with exogenous PGE2 in the same concentration of that found in the infected HOK supernatants.The collective results indicated that PGE_2_ produced by HOK can in turn impact the growth and adherence of *S*. *aureus*.

## Discussion

*S*. *aureus* is the main pathogen of many oral infections among which some can threaten patients’ life [4,5]. Thus, studying the behavior of *S*. *aureus* during the infection is helpful to better understand the mechanism in which *S*. *aureus* causes inflammation. In the present study, we demonstrated that *S*. *aureus* can induce COX-2 transcription and increase PGE_2_ production in oral epithelial cell line HOK. PGE_2_ promotes the growth of *S*. *aureus* and the binding of *S*. *aureus* to fibronectin. Importantly, we demonstrated that PGE_2_ facilitates the adherence of *S*. *aureus* to the oral epithelial cell. So far as we know, our study provides the first evidence for the facilitated role of PGE_2_ in *S*. *aureus* adherence.

Previous studies have indicated that COX-2 expression is increased in some cases of inflammation and cancer [11,13–15]. Increased level of PGE_2_ has been detected in the head and neck squamous cell carcinoma [54–56]. Report by Dimitrios Moraitis indicated that levels of COX-2 are increased by activating epidermal growth factor receptor (EGFR) in the oral mucosa of active smokers versus never smokers [16]. Here in this study, we first showed that *S*. *aureus* up-regulated COX-2 transcription and PGE_2_ production by normal oral epithelial cell line HOK. Consistent with this finding, increased PGE_2_ levels resulted from *S*. *aureus* infection were detected by Pérez-Novo in nasal tissue fibroblasts [17] and by Somayaji in murine osteoblasts [18]. Considering that PGE_2_ synthesis is involved with several enzymes, we thus used NS-398, a COX-2 specific inhibitor [57,58], to establish the role of COX-2 with respect to production of PGE_2_ during *S*. *aureus* infection of oral epithelial cell. After being treated with NS-398, both uninfected and *S*. *aureus*-infected HOK cells produced a significantly attenuated level of PGE_2_, which remarkably lower than that by cells without NS-398 treatment. These results indicate that *S*. *aureus* can increase COX-2 derived PGE_2_ production by HOK.

Intensive researches on PGE_2_ make the paradox and versatility of PGE_2_ function established. However, few studies hitherto have directly addressed the effects of PGE_2_ on bacterial pathogens. Recently Jan Krause reported that PGE_2_ produced by *C*.*albicans* displayed a stimulatory effect on the growth of *S*. *aureus* [37]. Consistently, we in this study found that both the purified and the produced PGE_2_ promoted *S*. *aureus* growth and biofilm formation at the experimental concentration. The observation that supernatant from HOK treated with NS-398 and infection failed to stimulate *S*. *aureus* growth and the addition of PGE_2_ reversed the facilitated effect that inhibited by NS-398 on *S*. *aureus* growth further confirmed that the enhanced growth of *S*. *aureus* is resulted from the PGE_2_ produced by HOK cells.

Fibronectin is a multifunctional extracellular matrix that plays a central role in cell adhesion and in the attachment of varies of microorganisms to human tissues [59,60]. *S*. *aureus* is the first bacterium shown to bind to fibronectin [61]. In this study, the binding of *S*. *aureus* to the fibronectin-coated surfaces is remarkably more than that to the uncoated surfaces, confirming the promotion of fibronectin to *S*. *aureus* attachment. Furthermore, our study first indicated that PGE_2_ significantly facilitated the ability of *S*. *aureus* to adhere to purified fibronectin, which manifested as the increased number of attached bacterial cells and the higher adhesion force value in the PGE_2_-treated *S*. *aureus* group than that in other control groups. Besides, the facilitated effect of PGE_2_ on the adhesion of *S*. *aureus* was also observed in the adherent assay to the oral epithelial cell line HOK. Through incubating *S*. *aureus* in absence of cells in the same condition during the adherent assay, we testified that the facilitation of PGE_2_ to attachment is not resulted from its growth promoting (S1 Fig).

The binding of *S*. *aureus* to fibronectin is mediated by cell-wall anchored fibronectin-binding proteins which encoded by two genes of *fnbpA* and *fnbpB*. According to study by Greene et al, the double mutant of *fnbpA* and *fnbpB* in *S*. *aureus* displayed severely impaired adherent ability to coverslips obtained from tissue cages implanted [41]. In this study, the transcriptional level of *fnbpB* mRNA was remarkably up-regulated by PGE_2_ both in the early and in the middle exponential phase. However, in the both phases, no change was observed in *fnbpA* transcription between the PGE_2_-treated and un-treated *S*. *aureus*. The transcriptional results of *fnbpA* and *fnbpB* indicated that PGE_2_ can in part regulate the transcription of genes coding for fibronectin-binding proteins.Several observations supported that *S*. *aureus* colonization is significantly higher in some cancer patients than that in the healthy [62–64] and COX-2 appears as frequently upregulated in tumor cells [65,66]. Take the Cutaneous T-cell lymphomas (CTCL) as an example, COX-2 expression has recently been proven in CTCL cells, and treatment with the selective COX-2 inhibitor celecoxib resulted in decreased cell growth and viability [66]. Meanwhile, as Nguyen and Talpur reported, patients with CTCL have a significantly higher rate of *S*. *aureus* than the general population [42,67]. Accordingly, our findings, in the present study, that PGE_2_ facilitates the growth and adherence of *S*. *aureus* supplies a reasonable presumption that the high rate of *S*. *aureus* colonization in cancers may in part attribute to the promoted impacts of PGE_2_ to the pathogen. Furthermore, based on the findings from this study and the study by Dimitrios Moraitis who reported that PGE_2_ production was increased in the oral mucosa of smokers [16], it is conceivable that the elevated PGE_2_ levels may cause a higher risk of *S*. *aureus* colonization in the oral mucosa of smokers. Thus, an active protection from *S*. *aureus* infection is essential for smokers.

Through binding to fibronectin which is simultaneously bound to integrin α5β1, *S*. *aureus* can be internalized into host cells. In the present study we also showed that PGE_2_ displayed an inhibitory role in the invasion of *S*. *aureus* to HOK cells, contrary to the facilitated impact on the adherence. The inconsistent roles of PGE_2_ in the attachment and invasion of *S*. *aureus* may due to the complicated mechanism by which *S*. *aureus* invades to cells. For example, observed evidence indicated that although α5β1 is expressed ubiquitously on human cells, the invasion level varies largely between host cells of different tissues [38,39,53,68,69]. Additionally, previous studies demonstrated that fibronectin promoted the binding of bacterial to polymorphonuclear leukocytes and macrophages but it didn’t facilitate ingestion or killing of the microorganisms [70,71]. Thus, the ability of *S*. *aureus* to use PGE_2_ to enhance its binding to fironectin is of some benefit to its parasites. Besides, as a pro-inflammatory factor, PGE_2_ was secreted by host cells to activate innate immune system to defend the pathogens impair [20,72,73]. Thus, the inhibition of PGE_2_ to *S*. *aureus* invasion demonstrated the defense reaction of the host to the harmful stimulation by such pathogen.

According to previous reports, chronic inflammation was suggested to promote cancer development by inducing the COX-2/PGE_2_ pathway and activating NF-κB and Stat3 signals [33,34]. This can be confirmed by the findings that regular use of non-steroidal anti-inflammatory drugs (NSAIDs) can reduce risk of gastrointestinal cancer and the growth of head and neck cancer [74–76] through blockading COX-1 and COX-2 activities, which subsequently suppresses prostaglandin, including PGE_2_, biosynthesis. In view of our present observations that *S*. *aureus* increases PGE_2_ production by HOK and PGE_2_ in turn facilitates the growth and adherence of *S*. *aureus*, further studies seeking for the probable mediating roles of COX-2/PGE_2_ pathway in the relationship between inflammation and cancer can be conducted, which may suggest COX-2/PGE_2_ axis targeting strategies for the prevention and treatment of inflammation and cancer diseases.

## Conclusion

To fully realize the potential of PGE_2_ targeting in the therapy of inflammation and cancer, sufficient investigations on the versatility of PGE_2_ are considerably essential. Here in this study, we first confirmed that *S*. *aureus* can up-regulated the COX-2 transcription and increased PGE_2_ production by the normal oral epithelial cell line HOK. Using the purified PGE_2_ and the supernatants, we found that in case of infection, *S*. *aureus* can intellectually take advantage of the surrounding PGE_2_ to increase its growth and adherence. These findings revealed a new look to the pathogenic mechanism of *S*. *aureus* and may lead to new therapeutic strategies with higher potency and improved selectivity.

## Supporting information

S1 Fig The number of *S*. *aureus* before and after incubated in DMEM for 45 min. Before incubated in DMEM, the initial number of *S*. *aureus* [*S*. *aureus* (before)] and *S*. *aureus* with PGE_2_ treated [*S*. *aureus* +PGE_2_ (before)] used for adhesion and invasion assay were approximately 2.63×10^7^ CUFs /mL and 2.57×10^7^ CUFs/mL. After treated in DMEM for 45 min, the numbers for them were 2.38×10^7^ CUFs/mL [*S*. *aureus* (after)] and 2.25×10^7^ CUFs/mL [*S*. *aureus* + PGE_2_ (after)] respectively. There was no significantly difference between all the groups.
(TIF)
